# Critical considerations for targeting colorectal liver metastases with nanotechnology

**DOI:** 10.1002/wnan.1588

**Published:** 2019-09-30

**Authors:** Usman Arshad, Paul A. Sutton, Marianne B. Ashford, Kevin E. Treacher, Neill J. Liptrott, Steve P. Rannard, Christopher E. Goldring, Andrew Owen

**Affiliations:** ^1^ Department of Molecular and Clinical Pharmacology University of Liverpool Liverpool UK; ^2^ Department of Molecular and Clinical Cancer Medicine University of Liverpool Liverpool UK; ^3^ AstraZeneca, Advanced Drug Delivery, Pharmaceutical Sciences, R&D Macclesfield UK; ^4^ AstraZeneca, Pharmaceutical Technology and Development Macclesfield UK; ^5^ Department of Molecular and Clinical Pharmacology, Materials Innovation Factory University of Liverpool Liverpool UK; ^6^ Department of Chemistry, Materials Innovation Factory University of Liverpool Liverpool UK; ^7^ MRC Centre for Drug Safety Science, Department of Molecular and Clinical Pharmacology University of Liverpool Liverpool UK

**Keywords:** colorectal cancer, drug delivery, liver metastases, nanomedicines, tumor biology

## Abstract

Colorectal cancer remains a significant cause of morbidity and mortality worldwide. Half of all patients develop liver metastases, presenting unique challenges for their treatment. The shortcomings of conventional chemotherapy has encouraged the use of nanomedicines; the application of nanotechnology in the diagnosis and treatment of disease. In spite of technological improvements in nanotechnology, the complexity of biological systems hinders the prospect of nanomedicines being applied in cancer therapy at the present time. This review highlights current biological barriers and discusses aspects of tumor biology together with the physicochemical features of the nanocarrier, that need to be considered in order to develop effective nanotherapeutics for colorectal cancer patients with liver metastases. It becomes clear that incorporating an interdisciplinary approach when developing nanomedicines should assure appropriate disease‐driven design and that this will form a critical step in improving their clinical translation.

This article is characterized under:

Therapeutic Approaches and Drug Discovery > Nanomedicine for Oncologic Disease

## Graphical abstract

A fundamental understanding of the tumor biology and its physiological features is necessary for the design and development of effective nanomedicines to target cancers.

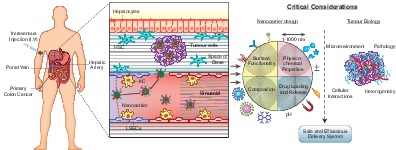

**ABBREVIATIONS**
ASGPR: asialoglycoprotein receptor
CRC: colorectal cancer
DC: dendritic cell
ECM: extracellular matrix
EPR: enhanced permeability and retention
HSC: hepatic stellate cell
KC: Kupffer cell
LSEC: liver sinusoidal endothelial cell
LYVE: lymphatic vessel endothelial hyaluronan receptor
mCRC: metastatic colorectal cancer
MPS: mononuclear phagocytic system
NK: natural killer cell
NP: nanoparticle
PEG: polyethylene glycol

## 1 INTRODUCTION

Colorectal cancer (CRC) is the third most common cancer worldwide, with more than 1.8 million new cases diagnosed in 2018 (10.2% of the total number of cancers diagnosed) (Bray et al., 2018). Metastatic disease often occurs for patients with CRC and the liver is primarily involved. At diagnosis, 14–20% of patients present with hepatic metastases (synchronous), and up to a third of patients will subsequently develop hepatic metastases (metachronous) during the course of their disease (Adam et al., 2015). Survival is greatly dependent on the stage of the cancer and the 5‐year overall survival for metastatic CRC (mCRC) remains low; less than 10% in recent reports (Cancer Research UK, 2018). Currently, various treatments are available for mCRC, including surgical resection, chemotherapy, biological therapies, radiotherapy and ablative techniques. Despite recent progress in surgical techniques in oncology, only 25% of patients are amenable to resection, which is considered to be the best option for long‐term survival. Alongside this, adjuvant chemotherapeutic agents in clinical practice have played an important role in reducing morbidity and enhancing quality of life. However, a major concern regarding systemic chemotherapy is their nonspecific distribution within the body, resulting in severe hematological, renal and hepatic toxicities. Other factors constraining the clinical effectiveness of such agents include: poor solubility, unfavorable pharmacokinetic profiles, drug metabolism, the development of multidrug resistance, accumulation in normal cells and suboptimal penetration into tumor tissues (Senapati, Mahanta, Kumar, & Maiti, 2018).

Over the last decade, considerable progress has been made in the field of nanotechnology. The development of a wide range of these technologies, varying in size, shape, composition and coatings, is altering the scientific landscape in relation to disease treatment and prevention (Singh & Lillard, 2009). As such, researchers are substantially exploring the use of these systems in preclinical and clinical settings. The area of nanoparticle (NP)‐based drug delivery receives much attention, as it provides opportunities to transform cancer treatment. Some key advantages of NP drug delivery include longer circulation half‐lives, improved pharmacokinetics, and targeted delivery decreasing off‐target toxicity (Parveen, Misra, & Sahoo, 2012). Despite the positive implications of NP drug delivery, only a small number have been approved and are currently being used in clinical settings. Several reports have indicated that suboptimal NP delivery into tumors is negatively impacting their clinical translation (Hare et al., 2017). The presence of multiple physiological barriers restricts the ability of NPs to successfully accumulate at their intended disease sites. Overcoming the difficulty of delivering NPs into solid tumors, features of the tumor microenvironment as well as nonspecific toxicity, present other major delivery challenges. Therefore, a greater fundamental understanding of the biological systems that prevent efficacy and uniform delivery of NPs into tumors is needed. This in turn will allow the development of strategies that enhance targeting and subsequently improve patient outcome.

### 1.1 CRC liver metastasis

Typically, CRC starts as an abnormal growth, termed an adenomatous polyp, on the epithelial surface of the bowel. Mutations that are common include alterations in tumor suppressor genes (p53 and APC), DNA repair genes (hMLH1 and hMSH2) and oncogenes (K‐ras and c‐Myc) (Hanahan & Weinberg, 2011; Kuipers et al., 2015). The accumulation of mutations gives abnormal cells within the colonic epithelium a selective advantage in terms of survival and proliferation. Consequently, the progressive accumulation of subsequent somatic mutations leads phenotypically to the formation of an adenomatous intermediate, ultimately leading to carcinoma.

Metastasis, initially described by Paget in his “seed and soil” hypothesis, is a complex multistep process that involves dissemination of tumor cells to distant organs. The liver is the main site of metastatic disease for many cancers, particularly CRC where it continues to be a major cause of cancer‐related death (Engstrand, Nilsson, Stromberg, Jonas, & Freedman, 2018). According to guidelines from the Union for International Cancer Control, these CRCs are classified as Stage IV (Brierley, Gospodarowicz, & Wittekind, 2017). The liver is the central metabolic organ and its unique architecture supports functions such as protein biosynthesis, host defense and detoxification of endo‐ and xenobiotics. Its dual blood supply (i.e., the hepatic artery supplies oxygenated blood and deoxygenated blood is provided via the hepatic portal vein) anastomose at the point of entry into the sinusoids and perfuse the parenchymal cells, before being drained by the hepatic veins into the inferior vena cava. However, features of the liver such as its anatomic position and histological architecture make it more susceptible to metastasis. Extensive branching of portal vessels into liver sinusoids slows down microcirculation, assisting mechanical arrest of circulating tumor cells. Extravasation of tumor cells is less restricted as the hepatic vasculature is fenestrated and a distinct sub‐endothelial basement membrane is absent. In addition, tumor cells are conveniently provided with access into the liver microvasculature via the hepatic portal vein (Gupta & Massague, 2006; Wray, Shah, Berman, & Ahmad, 2009).

**Figure 1:**
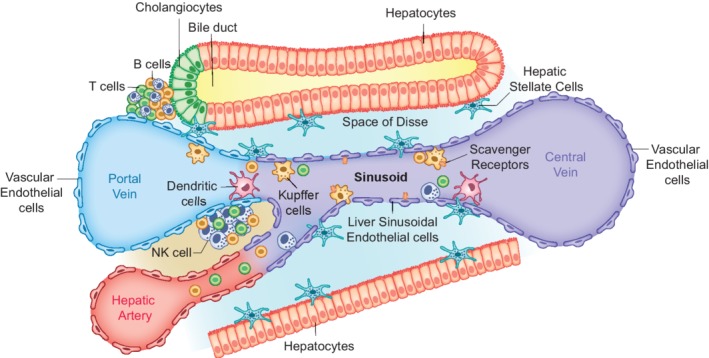
Schematic overview of a liver lobule highlighting the different hepatic cells. Oxygenated blood from terminal branches of the portal vein and hepatic artery merge upon entry into the liver sinusoids and drains into the central vein. The sinusoids are surrounded by fenestrated liver sinusoidal endothelial cells, which form a discontinuous endothelium that allows for bidirectional metabolic exchange. Kupffer cells, the specialized macrophages of the liver, are located in the lumen of the sinusoids and their primary function involves the removal of particulates from the portal blood. Hepatic stellate cells are positioned in the space of Disse and play a key role in the production of growth factors and cytokines. Hepatocytes, the functional unit of the liver, are arranged as interconnecting sheets of cells that surround the sinusoids. Bile produced by hepatocytes is collected into the bile ducts via the bile canaliculi. Cholangiocytes are the epithelial cells found lining the bile ducts

Prior to the onset of metastasis, tumor cells must detach themselves from the primary tumor. To accomplish this, tumor cells acquire an invasive phenotype (epithelial to mesenchymal transition) by reducing adhesion to adjacent cells, allowing migration into the vascular‐rich stroma. Circulating tumor cells are able to enter the liver through both vascular entry ports. The inferior and superior mesenteric veins and the portal vein, compose the foremost metastatic routes (Paschos, Majeed, & Bird, 2014; Wigmore, Madhavan, Redhead, Currie, & Garden, 2000). Once in the liver, they encounter the microenvironment of the hepatic sinusoid, which is composed of a diverse population of host cells that are specialized to carry out the multiplicity of hepatic functions, namely the liver sinusoidal endothelial cells (LSECs), Kupffer cells (KCs), hepatic stellate cells (HSCs), pit cells also called natural killer cells (NK) and hepatocytes, as shown in Figure 1 (Van den Eynden et al., 2013). The interaction of the tumor cells with hepatic sinusoidal and extrasinusoidal cells and their adaptation to the microenvironment governs their fate and the subsequent establishment of a metastasis within the liver. The aforementioned resident cells of the liver possess both antimetastatic and pro‐metastatic properties. LSECs can induce apoptosis of the circulating tumor cells by releasing nitric oxide and generating reactive oxygen species. Other antitumor responses include KC‐mediated phagocytosis and NK cell‐induced apoptosis, that is, through the release of cytotoxic granules containing perforin and granzymes (Lorenzo‐Herrero et al., 2018). Circulating tumor cells are able to evade these tumoricidal mechanisms by attaching to LSECs and transmigrating into the space of Disse (Milette, Sicklick, Lowy, & Brodt, 2017). The plethora of pro‐inflammatory cytokines produced during this process, causes activation of quiescent HSCs. This triggers the deposition of Type I and IV collagen and fibronectin, providing a scaffold for endothelial cell migration, angiogenesis and the framework for the establishment of micrometastases. Additionally, matrix metalloproteinases produced by KCs assist this process. Within the parenchyma these metastatic tumor cells can co‐opt existing vessels or trigger neoangiogenesis, to gain access or establish a blood supply. Furthermore, the presence of tumor cells within the liver can activate specific T‐cell‐mediated immune responses, restraining metastatic expansion through different cytolytic mechanisms (Brodt, 2016; Paschos et al., 2014). The tumor cells can evade such immune responses via co‐inhibitory molecules (PD‐1 and CTLA‐4), causing inhibition of effector T cell functions and adopting a state of immune tolerance. Subsequent proliferation and expansion ensues, leading to a transition from micrometastases to larger macroscopic metastases. However, not all tumor cells that survive have a capacity to proliferate, thus they may persist as micrometastases. These examples indicate that mCRC cells are capable of adapting within the hepatic microenvironment, improving their chances of survival and generating metastatic foci.

A combination of cytotoxic drugs with biologic agents in mCRC has become first‐line treatment, extending both progression‐free survival and overall survival (Cremolini et al., 2015). The most common chemotherapy regimens consist of FOLFOX and FOLFIRI—folinic acid, 5‐fluorouracil and oxaliplatin or irinotecan. Biological small molecule inhibitors are increasingly incorporated in treatment regimens and currently include: epidermal growth factor receptor inhibitors (cetuximab and panitumumab); tyrosine kinase inhibitors (erlotinib, gefitinib and lapatinib); and vascular endothelial growth factor inhibitors (aflibercept, bevacizumab, ramucirumab and regorafenib). Successfully targeting metastases has proven to be a challenging process. Difficulties are encountered in overcoming and navigating past biological barriers to target malignant cells within sites of metastasis. Continuous research in understanding the mechanisms underlying metastasis is opening opportunities for different treatments to be developed. Targeted drug delivery using nanotechnology‐enabled products offers previously unachievable opportunities to treat metastatic cancers.

### 1.2 Nanotechnology for drug delivery

Nanotechnology in medicine is increasingly being viewed as a promising tool that will eventually surpass conventional chemotherapy. A number of different nanocarriers are being investigated as controlled drug delivery vehicles for cancer therapy. These include carbon nanotubes, dendrimers, liposomes, micellar systems, NPs, and synthetic polymers (Mody, Tekade, Mehra, Chopdey, & Jain, 2014). These nanosized drug carriers offer a versatile platform to which many properties can be added and modified, with the overall aim of improving therapeutic effectiveness and specificity. Several of these nanomedicines have been approved for use in clinical practice (Table 1).

**Table 1:** Selected nanomedicines that have been approved or are in clinical development

Name	Particle type/drug	Application/indication	Approval year/phase	Advantages	References
Doxil®/Caelyx® (Janssen)	Liposomal Doxorubicin (PEGylated), ~100 nm	Ovarian cancer, HIV‐associated Kaposi's sarcoma, multiple myeloma	FDA (1995), EMA (1996)	Enhanced circulation time and up to six times more effective than free Dox	Barenholz, 2012
Myocet® (Teva UK)	Liposomal Doxorubicin (non‐PEGylated), 150–250 nm	Metastatic breast cancer	EMA (2000)	Better toxicity profile than free Dox (decreased occurrence of cardiac events and congestive heart failure)	Anselmo and Mitragotri, 2016; Teva UK, 2019
Onivyde® (Ipsen Biopharmaceuticals)	Liposomal Irinotecan (PEGylated), 80–140 nm	Metastatic pancreatic cancer	FDA (2015)	Prolonged circulation and reduced gastrointestinal toxicity	Ipsen Pharma, 2019; Tran, DeGiovanni, Piel, and Rai, 2017
Genexol® PM (Samyang Biopharmaceuticals)	Polymeric micelle formulated—Paclitaxel (PEG‐PLA), 20–50 nm	Breast cancer, lung cancer, ovarian cancer	South Korea (2007)	Enhanced tumor distribution, reduced toxicity present, cremophor‐free	Lee et al., 2008; Samyang Biopharm, 2019)
VYXEOS® (Jazz Pharmaceuticals)	Liposomal Cytarabine–Daunorubicin (non‐PEGylated), ~100 nm	Acute myeloid leukemia	FDA (2017), EMA (2018)	Improved overall survival	Jazz Pharmaceuticals, 2019
NK012 (Nippon Kayaku)	Polymeric micelle of SN‐38 (PEG‐PGA), ~20 nm	Small cell lung cancer, metastatic colorectal cancer	Phase II		Kayaku, 2019; Nakajima et al., 2008; Takahashi et al., 2010
CPX‐1 (Jazz Pharmaceuticals)	Liposomal formulation of Irinotecan and Floxuridine, 110 nm	Colorectal cancer, advanced solid tumors	Phase II		Batist et al., 2009
Onzeald™ Etirinotecan Pegol (Nektar)	Polymer drug conjugate of Irinotecan (PEGylated)	Metastatic breast cancer, ovarian cancer, colorectal cancer	Phase III		Hoch, Staschen, Johnson, and Eldon, 2014; Nektar, 2019
NLG207 formerly CRLX101 (NewLink Genetics)	Nanoparticle‐drug conjugate containing Camptothecin (Cyclodextrin‐PEG), ∼20–30 nm	Ovarian cancer, renal cancers, small cell lung cancer	Phase II		Svenson, Wolfgang, Hwang, Ryan, and Eliasof, 2011
AZD2811 (AstraZeneca)	Polymeric nanoparticle containing an aurora kinase B inhibitor (PEG‐PLA), 80–130 nm	Advanced solid tumors, hematological tumors	Phase II		Ashton et al., 2016; AstraZeneca, 2019
MTL‐CEBPA (MiNA Therapeutics)	SMARTICLES®‐based liposomal nanoparticle encapsulating CEBPA‐targeting saRNA	Liver cancer	Phase I		MiNA Therapeutics, 2019
Promitil® (LipoMedix Pharmaceuticals)	Liposomal Mitomycin‐C (PEGylated), 100 nm	Advanced colon cancer, solid tumors	Phase I/II		LipoMedix, 2019; Tahover et al., 2018

NPs are submicron‐sized particles with diameters ranging from 10 to 1,000 nm (Parveen & Sahoo, 2008). NP‐based systems possess unique physicochemical properties and their size, shape and surface properties can be tuned to modify the fate of both the NP and loaded drug (Shi, Kantoff, Wooster, & Farokhzad, 2017). Hence, the application of NPs in cancer therapy has been explored more widely than in any other disease, and their potential for optimizing the pharmacokinetics of chemotherapeutics and selectively accumulating high concentrations of cytotoxic agents within tumors have been reported (J. Cheng et al., 2007; Jain & Stylianopoulos, 2010; Koo et al., 2011; Senapati et al., 2018). There are a large variety of NPs being developed, namely lipid‐based, polymer‐based, and carbon‐based. Drugs can be loaded in/onto NPs by various means, such as physical entrapment (encapsulating anticancer drugs within their cores), covalent linking or surface attachment, that is, polymeric micelles and polymer conjugates. As such, NPs designed to preserve the drug in its active conformation could provide significant clinical benefit. Their surfaces can be additionally modified to incorporate specialized coatings or long chain polymers (polyethylene glycol, PEG) to impart increased stability and an enhanced circulatory half‐life (Knop, Hoogenboom, Fischer, & Schubert, 2010). Attaching targeting moieties including antibodies, nucleic acids, peptides, recombinant proteins and aptamers can further improve their selective accumulation (Rosenblum, Joshi, Tao, Karp, & Peer, 2018). On top of the mentioned physiological advantages of NPs, the tumor's pathophysiology offers ample opportunity for NP‐based drug delivery. In comparison to healthy tissues, solid tumors exhibit a morphologically irregular and abnormally acidic microenvironment. As a result, NPs tend to preferentially accumulate and localize within the tumor microenvironment, due to the hyperpermeable vasculature and dysfunctional lymphatic drainage within tumors—referred to as the enhanced permeability and retention (EPR) effect (Kobayashi, Watanabe, & Choyke, 2014; Matsumura & Maeda, 1986). Despite the impressive versatility and preclinical potential of NPs, very few NPs progress to the clinical arena. This review highlights the biological barriers and challenges that NPs encounter when targeting CRC liver metastases. Better understanding of these challenges will enable the rational design of fit‐for‐purpose materials, better able to produce beneficial clinical outcomes.

## 2 ACCESSING THE LIVER

### 2.1 The mononuclear phagocyte system

Systemic delivery via intravenous injection is the only route that has been successfully employed to deliver therapeutic NPs for cancer. Generally, complex NP therapies involving nanocarrier systems are difficult to deliver via other routes because they do not readily cross biological barriers in an intact form. Intravenous delivery is generally considered to be reliable and minimally invasive for cancer therapy, but NPs are subject to numerous physiological barriers within the circulatory system itself. The mononuclear phagocytic system (MPS), consisting of phagocytic cells located within organs including the bone marrow, spleen, liver and lymph nodes, sequesters and clears NPs from the systemic circulation (W. Jiang, von Roemeling, et al., 2017). Numerous serum proteins, in particular, opsonization by complement factors (C3, C4, and C5), fibrinogen, albumin, apolipoprotein and immunoglobulins, are adsorbed onto the surface of circulating NPs (Nguyen & Lee, 2017). This typically triggers the recognition of opsonized NPs by specialized receptors, resulting in NP clearance from circulation by the MPS. Examples of phagocytic cells comprising the MPS include blood circulating monocytes, splenic red pulp and marginal zone macrophages, hepatic KCs and LSECs, along with bone marrow macrophages (Davies, Jenkins, Allen, & Taylor, 2013; Y. N. Zhang, Poon, Tavares, McGilvray, & Chan, 2016). Given that premature elimination from the circulation obstructs NPs from accumulating in tumors, research has focused on the design of NP surface chemistries to minimize clearance via the MPS.

The most common technique involves functionalization of the NP surface with PEG, through a process called PEGylation. PEG molecules form a closely associated hydrating layer around the NP, impeding protein adsorption and subsequent clearance. In doing so, NPs are said to gain a “stealth” attribute and can remain in circulation for longer periods of time, increasing their chances of reaching target sites. This feature was best demonstrated by Doxil, whereby PEGylation extended the half‐life from minutes to hours (Gabizon, Shmeeda, & Barenholz, 2003). The impact of PEGylation varies for NPs differing in shape, size, and surface charge. PEGylation of smaller NPs results in a higher surface PEG density (larger hydrodynamic volume), meaning they are more readily able to evade clearance by the MPS (Alexis, Pridgen, Molnar, & Farokhzad, 2008; Walkey, Olsen, Guo, Emili, & Chan, 2012). To specifically study the effect of surface charge on MPS uptake, K. Xiao et al. (2011) were able to demonstrate that NPs with high negative or positive charges were taken up by murine macrophages in vitro as well as in vivo. Other surface modifications have included zwitterionic ligands, hydrophilic sugar coatings (dextran10) and the use of biological proteins (INNO‐206), but PEG remains the most widely used conjugation technique. Several PEGylated NPs loaded with chemotherapeutic agents have displayed selective tumor accumulation and enhanced inhibition in various xenografted models of CRC liver metastases (B. L. Chen et al., 2016; Luo et al., 2018; Pohlen, Buhr, & Berger, 2011; H. Zhang et al., 2015). Such results demonstrate the advantages of PEGylated over non‐PEGylated drug delivery systems for cancer therapy. Of note, EZN‐2208 is a PEGylated prodrug loaded with SN‐38 that showed prolonged circulation in various preclinical tumor xenograft models in comparison to free drug. PEG was attached in a manner that stabilized the drug, maintaining its active conformation (Gritli et al., 2016; Sapra et al., 2008). However, when given to patients with advanced CRC in a randomized clinical study, EZN‐2208 did not demonstrate superior efficacy in terms of overall survival in comparison to Irinotecan (Garrett et al., 2013). Possible reasons for this were thought to be due to poor tumor drug distribution and pharmacokinetics. Therefore, even after NPs have successfully evaded clearance by the MPS, they still need to be able to effectively extravasate towards and penetrate the tumor microenvironment.

### 2.2 Passive and active targeting of CRC liver metastases

**Figure 2:**
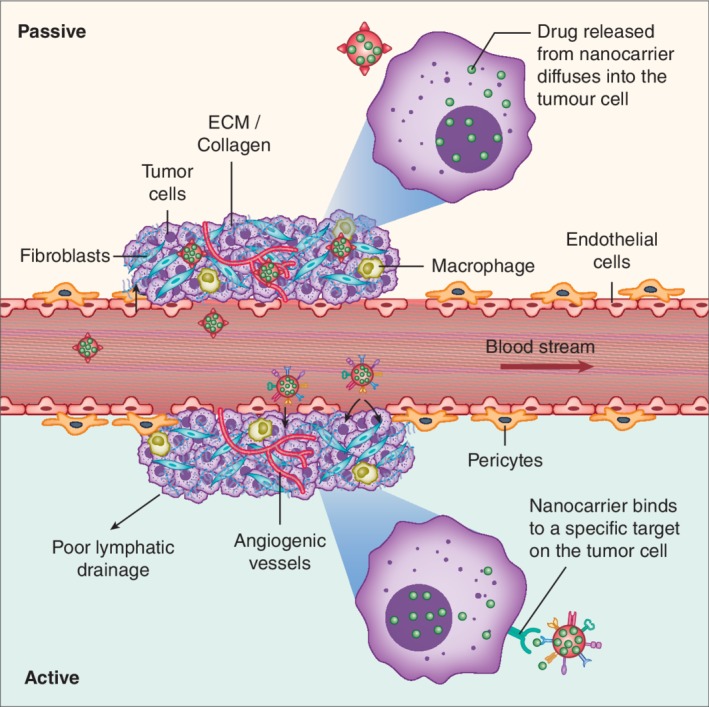
Schematic representation of passive and actively targeted nanoparticle (NP) delivery systems. By taking advantage of the enhanced permeability and retention (EPR) effect, nontargeted NPs are able to passively extravasate through the leaky vasculature and accumulate within the tumor (upper). Alternatively, the surface of NPs can be conjugated with targeting moieties to actively bind with a cell‐specific target (lower). This allows for enhanced cellular uptake inside the tumor

Alongside the MPS, both passive and active targeting play a key role in assisting NP delivery to tumor sites as depicted in Figure 2. Large fenestrae of 100–200 nm (increases to 400–600 nm in some liver diseases) and an absence of an organized basement membrane are features of the liver sinusoids (Aird, 2007). Hence they are able to facilitate passive liver targeting through the widely reported EPR effect. This effectively builds up a high local concentration of NPs in the perisinusoidal space of Disse, whereby diffusion towards the malignant tumor cells can occur. Since the EPR effect is evident within tumors, it results in the preferential uptake and retention of NPs (Maeda, Wu, Sawa, Matsumura, & Hori, 2000). Excessive pro‐angiogenic signaling within the tumor microenvironment leads collectively to the production of immature blood vessels that are heterogeneous, poorly perfused and disorganized. Leakage results from large inter‐endothelial cell junctions, favoring the retention of NPs. These mechanistic features underpin the EPR effect and have been greatly exploited as a strategy to passively deliver NPs into tumors. It was established that NK012, a polymeric micelle formulation of SN‐38, enhances its antitumor activity through the EPR effect. By using a VEGF‐secreting tumor model, significantly enhanced accumulation of NK012 within tumors was demonstrated, augmented by the hypervascularity and hyperpermeability induced by VEGF (Koizumi et al., 2006). Similarly, in a Phase II clinical trial the accumulation of the liposome, CPX‐1, in CRC lesions was attributed to EPR‐based accumulation (Batist et al., 2008; Goel et al., 2011; Golombek et al., 2018). Despite the benefits of passive targeting, major limitations still exist. Heterogeneity within tumors and between the primary and metastatic tumors, often causes the nonuniform delivery of NPs (Adua et al., 2017). Additionally, passive delivery of NPs can result in drug exposure to healthy tissues and cells, that is, passively targeted NPs release their payload into the tumor microenvironment rather than within tumor cells. This can reduce therapeutic effectiveness, promote drug resistance and promote off‐target toxicity (W. Jiang, von Roemeling, et al., 2017). A possible solution to overcome such drawbacks is to additionally actively target NPs.

Chemotherapeutic treatments for mCRC are not as effective as for other cancers, because of the difficulty of delivering drug at effective concentrations to the target site. In many cases, the failure to achieve effective doses at the tumor site leads to ineffective chemotherapy, cancer recurrence and reduced patient survival. A targeted approach may therefore be applied, to advance the pharmacological properties and reduce the toxicities associated with the treatment of mCRC. Active targeting requires the conjugation of recognition molecules that can promote retention of NPs specifically at the tumor site. It is generally achieved by targeting surface molecules or receptors overexpressed on either the tumor cells microenvironment or its vasculature. Careful selection of the targeting moiety is needed, and the target should be highly and preferentially expressed at the tumor site. Moreover, the targeting moiety should have strong and specific binding affinity to the intended target and be well suited to chemical modification by conjugation. However, to take advantage of this increased affinity, actively targeted NPs must first reach the target site. Effective passive targeting is therefore essential for these NPs to increase their accumulation and distribution at the tumor site. Recently, Sharma et al. demonstrated that NPs functionalized with folic acid displayed preferential in vivo uptake in colon tissue (Cisterna et al., 2016). Similarly in a mCRC experimental model, folate conjugated pRNA NPs were found to be specifically retained in liver metastases, without any untargeted uptake in other organs (Rychahou et al., 2013). Other investigations have shown that using hyaluronic acid as a targeting ligand on the surface of NPs, resulted in increased CRC targeting when compared to the nontargeted NPs (B. Xiao et al., 2015). Further, widely explored cellular targets for CRC include CD44, ASGPR, VEGFR, EGFR, TfR1, DR‐5 carcinoembryonic antigens, TAG‐72, integrin α5β1 and A54 peptide surface markers (Cohen & Margel, 2012; Schmid et al., 2014; Y. N. Zhang et al., 2016). Presently, methods to prime the tumor microenvironment are being reviewed to enhance NP delivery by normalizing the tumor microenvironment (Jain & Stylianopoulos, 2010; Khawar, Kim, & Kuh, 2015; Stylianopoulos, Munn, & Jain, 2018). Despite the enhanced efficacy demonstrated in various studies, factors including; immunogenicity and stability of the targeting moiety leading to premature NP clearance, uneven exposure to chemotherapy—bringing about tumor recurrence, drug resistance and loss of activity due to receptor‐mediated endocytosis, severely hinder the application of targeted NPs. Research conducted by Wilhelm et al. (2016) highlighted this point, as they showed that active targeting only slightly improves the number of NPs that are efficiently delivered to tumors.

Several passively targeted nanomedicines have received clinical approval starting with Doxil in 1995 (Table 1). In spite of the abundant success of actively targeted nanomedicines preclinically, very few have been approved, and include the antibody drug conjugates Adcetris, Besponsa, Kadcyla and Mylotarg (Dan et al., 2018; Rodrigues & Bernardes, 2018). Physiological barriers such as tumor penetration, relative hypoxia and endosomal escape continue to challenge the success of actively targeted NPs by effectively limiting their therapeutic benefit. The biological and physicochemical properties of NPs can also influence their distribution and accumulation in tumors (Bertrand, Wu, Xu, Kamaly, & Farokhzad, 2014). Added on to this, the present understanding of EPR effectiveness is limited due to the fact that most preclinical tumor models do not accurately represent solid tumors in humans, for example, tumor xenografts are rapidly growing and the EPR effect is more prominent. This could potentially inflate the benefits of NPs relying on the EPR effect (Prabhakar et al., 2013; Rosenblum et al., 2018). Moreover, there is insufficient patient‐based investigational data on the EPR effect itself, hence further clinically based research is required. In the case of actively targeting liver tumors, an additional stumbling‐block is selective and uniform cellular uptake into the tumor cells, while minimizing nonspecific effects on other healthy cell types. It is only under such circumstances that selective drug delivery can be achieved. Despite this, there is a clear trend in ongoing clinical trials and emerging studies towards the use of actively targeted therapeutics.

## 3 CELLULAR INTERACTIONS WITH HEPATIC CELLS

The interaction of NPs with the various cells located within the liver is a key factor in governing their fate. Though it is evident that NPs accumulate in the liver and this is influenced by their physicochemical properties, relatively little is understood about this hepatic distribution of NPs. Notably, parenchymal (hepatocytes and cholangiocytes) and nonparenchymal cells (KCs, LSECs, HSCs, macrophages, and liver infiltrating lymphocytes) have to be considered and sometimes avoided, to assist extravasation and targeting of NPs into the tumor microenvironment. Current data has shown that the velocity of the NP reduces up to 1,000‐fold once it enters the liver, enhancing the prospect of NP interactions with the hepatic cells (Tsoi et al., 2016).

KCs are a specialized population of macrophages, which account for the majority of phagocytic activity in the liver. They comprise about 2.5% of the total liver volume, which makes 8.5% of the total number of liver cells. KCs are positioned in the vascular space of liver sinusoids and are an important first‐line of innate immunity. They express a range of scavenger, complement, antibody and toll‐like receptors, allowing KCs to respond to danger signals. As part of the MPS, KCs are proficient at internalizing and removing circulating NPs, pathogens and associated molecules, through a process of phagocytosis and phagolysosomal degradation. KCs are plastic cells, expressing a polarized M1 pro‐inflammatory or M2 anti‐inflammatory phenotype, depending on the immune and metabolic environment they are exposed to (Tsoi et al., 2016). Together with their location, primarily at the periportal end of the sinusoids, KCs have been shown to rapidly sequester NPs (>100 nm in size) and studies have highlighted KCs to be the cell type primarily responsible for avidly sequestering NPs in the liver (Sadauskas et al., 2007). Moreover, within the liver microarchitecture the blood flow is significantly slower than in the systemic circulation, which increases the probability of nonspecific interactions of NPs with these cells. Other results demonstrated that KCs with an M2 phenotype preferentially uptake NPs, while it has been shown that negatively charged NPs are more likely to interact with KCs (MacParland et al., 2017). Many different strategies to avoid uptake by KCs have been reported in the literature. Crettaz et al. (2006) displayed that direct parenchymal injection of adenoviral vectors through intrahepatic injection were able to partially escape KCs, reducing toxicity and implying that the route of administration requires additional consideration. Moreover, increasing the length of PEG (30 kDa) allowed vectors to evade KC scavenging (Prill et al., 2011). However, approaches looking into depletion of KCs or impeding receptors mediating uptake, displayed little impact on the cell‐mediated accumulation of NPs (Park et al., 2016; Samuelsson, Shen, Blanco, Ferrari, & Wolfram, 2017). Such findings denote that other cells in the hepatic microenvironment may also play a contributing role in liver sequestration of NPs.

The LSECs make up approximately 3.3% of the total liver volume, which comprises 21% of the total number of liver cells. LSECs form a thin lining along the vasculature of the sinusoids. The presence of fenestrae and open pores separates blood from the hepatocytes. LSECs are actively endocytosing cells and they rely on scavenger receptors to remove an array of molecules from the bloodstream. Some of the major receptors involved include: mannose receptor, Fcγ‐receptor IIb2, collagen‐alpha receptor, and the hyaluronan (stabilin 1/2) scavenger receptor. The former two mentioned receptors are also expressed by KCs and alongside LSECs they work to sequester NPs in circulation. The interaction of NPs with these scavenging cells may vary due to a number of different factors, that is, physicochemical properties of the NP, opsonins adsorbed on the NP surface, cellular location of LSECs or KCs and the influence of the local microenvironment. The general consensus based on previous work is that smaller monodisperse NPs interact with LSECs, while NPs that are over 100 nm in diameter are cleared by KCs (Shiratori et al., 1993; Sorensen, Simon‐Santamaria, McCuskey, & Smedsrod, 2015). Despite this, further research is needed to understand the mechanisms of uptake and how this can vary. Recently, it has been established that the LSEC scavenger function rests largely on the ability of stabilin‐1 and stabilin‐2 receptors to mediate the recognition and uptake of NPs. This was observed in a zebrafish model whereby negatively charged NPs were readily taken up by SECs in a stabilin‐dependent fashion, and the use of dextran sulfate, a competitive inhibitor of stab2, blocked this NP–SEC interaction (Campbell et al., 2018; Sorensen et al., 2015). These same stabilin receptors on LSECS were targeted with miR‐20a‐loaded NPs and shown to reduce the pro‐metastatic role of LSECs in a murine mCRC model (Marquez et al., 2018). Furthermore, researchers have explored methods to reduce NP uptake by LSECs and it has been revealed that the conjugation of a second PEG layer on conventional PEGylated NPs considerably lengthens their time in circulation (Zhou et al., 2018).

Other cells such as hepatocytes and HSCs are capable of internalizing NPs, but less efficiently than KCs. Hepatocytes constitute 70–80% of the liver population and are organized into two unicellular layers that border the sinusoids. These cells represent an important physiologic pathway for NP processing and their capacity to internalize NPs means they are a potential site of toxicity (Gaiser et al., 2013). Hepatocytes are within the pathway for biliary excretion and NPs are taken up to facilitate their enzymatic breakdown or removal. Additionally, these cells are involved in the uptake of positively charged NPs but not their negatively charged counterparts. It was demonstrated that the opsonins apolipoprotein E and IgA influenced hepatocyte‐directed NP uptake, when bound to the positively charged NP surface (S. H. Cheng et al., 2012; Wang et al., 2015). Likewise, a size‐dependent effect was postulated, as 50 nm gold NPs showed enhanced hepatocyte targeting compared to larger counterparts (80–150 nm) (Y. N. Zhang et al., 2016). Further studies across material types are needed to confirm these size‐dependent effects. Given that direct tumor cell–hepatocyte interactions during liver metastasis have been observed, a wide variety of NPs have been designed to target hepatocytes (D'Souza & Devarajan, 2015; Longmuir, Haynes, Baratta, Kasabwalla, & Robertson, 2009; Y. N. Zhang et al., 2016). Many have utilized ligands to target the asialoglycoprotein receptor (ASGPR), which is expressed on hepatocytes, in the hope that its elevated expression provides an opportunity to specifically target these tumor cells. It should be noted however that healthy hepatocytes also typically express ASGPRs, presenting a risk that such targeted NPs may also accumulate in normal cells with adverse effects.

HSCs reside in the space of Disse between LSECs and hepatocytes. They represent the body's main store of vitamin A and amplify the inflammatory response by secreting cytokines following liver damage. Despite it being shown that phagocytic activity is indeed a characteristic, the peri‐sinusoidal location of HSCs suggests it is more than likely that the majority of NPs will be initially internalized by KCs and LSECs. Recent in vitro studies have proposed HSCs to be a fundamental component of the pro‐metastatic liver microenvironment. This is due to their ability to trans‐differentiate into highly proliferative and motile myofibroblasts, which have been implicated in supporting tumor growth (Kang, Gores, & Shah, 2011).

Liver‐associated lymphocytes encompass B cells, T cells, dendritic cells (DCs) and NK cells. They are located in the sinusoidal lumen, in contact with KCs and/or LSECs, but also dispersed throughout the parenchyma. While NK cells primarily play a role in host defense by inducing cell death in infected/damaged cells, antigen processing cells such as DCs process and present pathogens as well as exogenous proteins/peptides to adaptive effector cells including B cells and T cells. The latter are found bordering the portal triad and both have been shown to possess endocytic/phagocytic capacity and internalize NPs (Huq et al., 2016; Tsoi et al., 2016). DCs are predominantly situated in the portal regions and sporadically in the parenchyma. The latest studies revealed that DCs also actively internalize NPs due to their high phagocytic capabilities (Cruz et al., 2017; Jia et al., 2018). NK cells are also able to undertake process such as phagocytosis and reports have shown that they are capable of receptor‐mediated endocytosis (Peruzzi, Masilamani, Borrego, & Coligan, 2009). However, to what extent NK cells influence the biodistribution of NP in the liver is currently unknown and warrants further investigation. Direct immunosuppressive mechanisms have been demonstrated in patients with CRC liver metastases. This often involves suppressed NK cell activity and impairment of DC differentiation within the cancer, ultimately inhibiting T cell proliferation and the antitumor response (Legitimo, Consolini, Failli, Orsini, & Spisni, 2014; Pancione et al., 2014; Zumwalt & Goel, 2015). Recent successes in cancer immunotherapy has led to the design of NPs that are able to manipulate the immune response against tumors, that is, activated immune cells can home in on the tumor and exert their therapeutic effect. This contrasts with the current approach in designing cancer nanomedicines, with the focus primarily on avoiding interactions of NPs with the immune system, while enhancing NP delivery into tumors. Q. M. Xu et al. (2012) intravenously administered interleukin‐12‐loaded NPs for liver metastasis targeting in Balb/c mice. Following histological assessment of these livers, the NPs efficiently induced the recruitment and tumor infiltration of NK and T cells, causing a significant decrease in the number and volume of CRC liver metastasis foci compared with free interleukin‐12. Therefore, priming an antitumor immune response distant from the site of disease, has the potential for the safe treatment of CRC liver metastases.

To appropriately formulate effective strategies going forward, it is of high importance that interactions and distribution of NPs with the different cells types located within the liver be considered and understood. This is crucial from a therapeutic standpoint, since off‐target uptake of NPs could have deleterious consequences.

## 4 TRANSPORT TO CRC TUMOR CELLS IN THE LIVER

**Figure 3:**
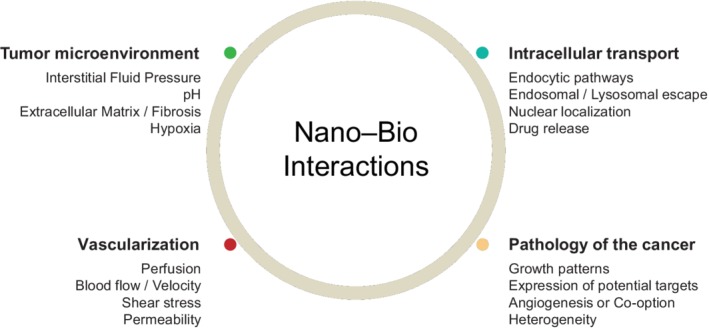
Overview of several key interactions involving nanoparticles within the tumor

Following their arrival at the tumor vasculature, NPs are able to extravasate into the tumor microenvironment. Once there, the NPs must navigate their way through the tumor interstitium including physical/extracellular barriers (interstitial space, extracellular matrix [ECM], and cell membranes), to access the tumor cells or specific intratumoral structures. The microenvironment in CRC liver metastases consists of a complex network of cells encompassing tumor cells, immune cells and soluble factors together with a variety of stromal cells including collagens and fibroblasts. Various histological growth patterns have been biologically described in CRC liver metastases; desmoplastic, pushing and replacement. Desmoplastic growth patterns are characterized by a rim of fibrotic stroma at the tumor–liver interface, which possesses a rich inflammatory infiltrate, high proteolytic activity and increased endothelial cell proliferation. In metastases with a pushing pattern of growth, the fibrotic stroma is lacking and the liver cells and structures appear to be compressed (Moro, Bozoky, & Gerling, 2018). Liver metastases with a replacement pattern grow without inducing hypoxia or angiogenesis. Instead they co‐opt the preexisting sinusoidal vasculature and have little effect on the existing liver architecture, that is, not causing inflammation and fibrosis (Kuczynski, Vermeulen, Pezzella, Kerbel, & Reynolds, 2019; Lazaris et al., 2018; van Dam et al., 2017; Van den Eynden et al., 2013). In contrast, metastases with a pushing and to a lesser degree desmoplastic growth pattern display signs of active, hypoxia‐driven angiogenesis. The metastatic growth patterns for CRC also vary in their invasive potential, as they differentially express proteases involved in the breakdown and remodeling of the ECM (Illemann et al., 2006, 2009). Thus, the features of the tumor as well as the various facets that compose the tumor vasculature and microenvironment may diminish the effects of NPs and need to be considered. These have been outlined below in Figure 3.

### 4.1 Vascularization and perfusion

As mentioned above, the tumor vasculature is tortuous and irregular leading to a compromised blood flow. This may further influence the site‐specific accumulation of NPs. Although the permeability of the tumor vasculature forms the basis of the EPR effect, it also allows excessive extravasation of blood constituents such as fluid and plasma macromolecules. Elevated fluid viscosity inside the tumor mass is therefore apparent, hindering the movement of NPs. Vascular perfusion inside tumors is heterogeneous and certain intratumoral regions can be hypoperfused due to a decreased blood flow caused by plasma leakage, in conjunction with blood vessel compression (Stylianopoulos et al., 2018). Recent findings have revealed that the levels of Type IV collagen are significantly higher inside liver metastases when compared to their primary tumors (Kai et al., 2019). In general, it is thought that liver metastases are poorly perfused in comparison to their primary tumor and their vascular permeability is limited (Tanei et al., 2016). Accordingly, NP delivery is limited as tumor cells are too distant from normal functioning vessels, ultimately restricting the distribution of therapeutic agents.

Previous research has shown that the hepatic artery supplies large metastatic tumors, while micrometastases (<2 mm) have been reported to be predominantly supplied by the portal venous system (Casillas et al., 1997). More recent observations have shown that vascularization also differs depending on the type of liver metastases. Due to vessel co‐option, replacement liver metastases are often in continuum with the sinusoidal blood vessels, whereas in desmoplastic liver metastases the capillaries connect to blood vessels of the arteriole (Lazaris et al., 2018; Van den Eynden et al., 2012). Further evidence revealing that replacement liver metastases co‐opt the preexisting vasculature is indicated in reports, as co‐opted vessels maintain expression of lymphatic vessel endothelial hyaluronan receptor (LYVE)‐1 (Stessels et al., 2004). On the other hand, desmoplastic liver metastases contained very few LYVE‐1‐expressing vessels, indicative of angiogenesis. This angiogenesis seen in desmoplastic liver metastases is distinguished by regions of high vessel density termed vascular hotspots. These newly formed blood vessels appear leaky and functionally impaired due to the presence of fibrin deposits. In contrast, replacement liver metastases display a small amount of proliferating endothelial cells, with no apparent signs of fibrin deposition (Stessels et al., 2004). The appearance of vessel co‐option could have important implications in the context of NP delivery. In this manner, liver metastases may incorporate “normal looking” vasculature, that is, less permeable and unlikely to display the EPR effect. Hence, NPs whose actions solely rely on the abnormal vasculature may be impaired and any strategies to normalize the tumor vasculature through antiangiogenic therapies may be hindered by drug resistance, as seen from previous studies (Emblem & Jain, 2016; Frentzas et al., 2016; Qian, 2013). More recently, it was highlighted that approximately two‐thirds of patients presented with mixed growth patterns (mixed phenotype of co‐opted vessels and angiogenesis) (van Dam et al., 2017). Therapeutic approaches that can suppress both angiogenesis and vessel co‐option may therefore be justified. In particular, specifically targeting the co‐opted vasculature by capitalizing on their altered protein expression via active targeting strategies may be a possibility (Roodink et al., 2010).

Drug delivery strategies need to ensure sufficient drug penetration/distribution is achieved, such that both the peripheral and central regions of the tumor are accessed. Some researchers have sought to manipulate the tumor vasculature through utilizing hyperthermia and growth factors, with the intent being to facilitate extravasation of NPs into the tumor microenvironment (Chatterjee, Diagaradjane, & Krishnan, 2011; W. Jiang, Huang, An, & Kim, 2015). This is exemplified by local hyperthermia (41°C) eliciting an increase in vascular permeability up to 10 μm in a variety of tumor models, allowing greater penetration into the tumor microenvironment (L. Li et al., 2013). For instance, ThermoDox (lyso‐thermosensitive liposomal doxorubicin technology) was combined with image‐guided radiofrequency ablation in a porcine model (Swenson et al., 2015). In the presence of heat, the amount of doxorubicin deposited increased and the treatment zone was enlarged. Despite this, no clinically meaningful benefit was found in patients with hepatocellular carcinoma in the Phase III HEAT study (Lencioni & Cioni, 2016). Similarly, a Phase I trial examined the combined treatment of thermosensitive liposomal doxorubicin and noninvasive focused ultrasound hyperthermia (39.5–43°C), with the latter being used to trigger drug release (Lyon et al., 2018). The study displayed enhanced intratumoral delivery in human liver tumors (patients investigated had solid primary or metastatic tumors), while also posing no additional safety concerns in comparison to standard chemotherapy alone. Hyperthermia itself is able to exert a cytotoxic effect and following combination with certain cytotoxic agents, synergistic effects are present (Jacquet, Averbach, Stuart, Chang, & Sugarbaker, 1998; Kusamura, Dominique, Baratti, Younan, & Deraco, 2008). Other reports have also shown hyperthermia to modify membrane permeability, drug uptake and drug penetration (Di Miceli et al., 2012; Hompes et al., 2012). A recent meta‐analysis displayed that patients with mCRC show a tendency towards increased median overall survival after cytoreductive surgery and hyperthermic intraperitoneal chemotherapy combined with resection of liver metastases, when compared to treatment with systemic chemotherapy (de Cuba et al., 2013). However, there are several challenges still facing the mentioned approaches, which include tumor heterogeneity and regional variation. The tumor vascular network remains a major obstacle in the vascular transportation of NPs.

### 4.2 The tumor microenvironment

The mCRC tumor microenvironment is a multifaceted dynamic system. Its regulation depends on the interactions between cellular (tumor cells and resident cells of the liver) and noncellular components (hypoxia, pH, signaling molecules, and ECM). Compared to normal tissue, the tumor microenvironment possesses numerous unique characteristics. It presents an acidic pH due to the Warburg effect and hypoxia is generated as cells residing deep in the tumor mass are deprived of oxygen because of inadequate vasculature, lack of nutrients and uncontrolled proliferation. In particular, hypoxia within CRC liver metastases has been observed at an average distance of 80 μm from the vasculature and it is postulated that large oxygen consumption is the principal contributing factor (van Laarhoven et al., 2006). Taking into consideration such features of the tumor microenvironment when designing NPs, may aid their delivery into tumors. Various NPs have been designed to utilize the acidic pH microenvironment to selectively trigger release. For instance, polymeric micelles with a pH‐sensitive component containing cell‐penetrating peptides were designed for the treatment of CRC (Bao et al., 2016). Following intravenous administration, findings revealed the destabilization of the polymer at lower pH (pH 6.8) allowed for enhanced action, both in vitro and in vivo, owing to improved targeting and cellular uptake. The hypoxia in tumors can also be employed to control drug release or activate prodrugs. Kulkarni et al. (2016) constructed lipid NPs containing a hypoxia‐sensitive component, which displayed a better penetration depth and cytotoxicity under hypoxic conditions. Similarly, hypoxia‐responsive doxorubicin NPs have been reported to selectively release drug under hypoxic conditions (Thambi et al., 2014). These NPs exhibited enhanced antitumor efficacy, higher toxicity to hypoxic cells and in vivo imaging showed them to successfully accumulate at the tumor site. The presence of improved penetration demonstrated the advantages offered by hypoxia‐activated chemotherapeutic delivery. Even with recent advances in the development of hypoxia‐responsive NPs, accessing hypoxic regions located deep inside the tumor remains an unmet challenge.

Another hurdle compromising transport of NPs is the high interstitial fluid pressure (IFP). IFP is elevated as a result of the abnormal vasculature, intratumoral lymphatic vessels that do not effectively drain interstitial fluid and a dense ECM. Moreover, it has been demonstrated in the literature that patients with CRC liver metastases, had a mean IFP 10 times above the IFP of normal liver tissues (Less et al., 1992). The IFP at regions close to the tumor margin is often normal, meaning that there is an outward pressure gradient (Jain, Tong, & Munn, 2007). Consequently, it can limit the ability of NPs to diffuse into the tumor interstitium, slow down their biodistribution, and hinder NP transport within the vasculature. In the worst case scenario, the IFP can cause intravasation of NPs back into the blood supply. To try and counteract this, researchers utilized gelatin‐modified cationic lipid NPs with the aim of reducing tumor IFP and subsequently improving drug delivery. It was demonstrated that delivery of imatinib caused a significant reduction in tumor IFP, when combined with docetaxel and quercetin in NPs (Gao et al., 2017). Likewise, the reduction of tumor IFP by eradicating fibroblasts promoted deeper chemotherapeutic penetration in HepG2 spheroids, thereby demonstrating that reducing tumor IFP could be beneficial for improving NP delivery (B. L. Chen et al., 2016). PEGPH20 is a therapeutic candidate currently being explored for improving local permeation, through the degradation of hyaluronan in the tumor microenvironment. This reduces tumor IFP, and PEGPH20 is thought to enable increased access for anti‐cancer therapeutics and immune cells (Halozyme, 2019). Its use in advanced pancreatic adenocarcinoma and metastatic breast cancer have shown encouraging results in terms of safety, overall survival and response rate, thereby prompting further studies (Gourd, 2018).

The ECM consists mainly of a cross‐linked network of collagen, elastin fibers, proteoglycans and hyaluronic acid. ECM proteins can interact with growth factors promoting cell migration and metastatic progression. The ECM is often more dense and highly cross‐linked within tumors. Patients with CRC liver metastases have shown to display abnormal ECM protein synthesis and degradation, with collagen turnover‐related proteins being upregulated (Van Huizen et al., 2019; Williamson, Sultanpuram, & Sendi, 2019). This may pose a significant barrier to the diffusion of NPs through the interstitium, causing drug release distant from the tumor cells. Within the tumor microenvironment trans‐differentiated HSCs, tumor‐associated macrophages and the KCs create a reactive tumor stroma by producing a plethora of remodeling factors, including the proteolytic matrix metalloproteinase enzymes involved in ECM turnover (Kang et al., 2011). This further impacts the composition, structure and elasticity of the ECM. These factors have to be accounted for, in order to obtain appropriate interstitial penetration and homogeneous drug distribution. In conjunction with this, combining locoregional therapy such as hepatic arterial infusion or transarterial chemoembolization with nanotechnology and the use of intratumor depots/implants for sustained release may overcome the limitations of the tumor biology. In a rat model of CRC liver metastases, Kauffels et al. (2019) were able to show that delivery of drug loaded into embolization particles (Irinotecan with EmboCept S) using hepatic arterial infusion led to significantly higher concentrations within the tumor when compared with systemic administration. Clearly, the application and toxicity of such delivery strategies requires further investigation.

It is still unknown to what extent the tumor microenvironment influences the different metastatic growth patterns and directs whether blood vessels are preexisting or newly formed. Such attributes of CRC liver metastases are not fully understood in humans, with research being primarily conducted in animal models. Experiments have suggested that the adopted growth pattern is strongly associated with the route of dissemination into the liver (Bugyik et al., 2016; Paku, Kopper, & Nagy, 2005; Paku & Lapis, 1993). In a mouse model of liver metastases, once tumor cells were delivered into the liver via the arterial system they displayed signs of angiogenesis, as seen by the presence of numerous small vessels. Whereas, entry into the liver via the portal vein corresponded with co‐option of sinusoidal vessels (Paku & Lapis, 1993). Mechanistic features of vascularization at early stages of CRC liver metastases in humans have not been completely described. It is believed that a number of elements such as the tumor cells, angiogenic factors (VEGF, HIF‐1α, E‐selectin and endothelin) and the host microenvironment influence this process. Published reports have pointed out that activated HSCs play a central role in the development of the vasculature (Paku et al., 2005; B. Xu, Shen, Cao, & Jia, 2013). They are thought to create a proangiogenic microenvironment and to be responsible for the recruitment and survival of endothelial cells. Researchers investigated changes that occur in intratumoral microvessels and microcirculation during the establishment of liver metastases in mice. Findings showed that liver metastases smaller than 520 μm were hypovascular, while those larger than 2000 μm showed an exclusively arterial blood supply. Increased alpha‐smooth muscle actin positive arterioles and levels of CD34 (marker of tumor neovascularization) were also seen (Archer & Gray, 1989; Joo et al., 2011; Liu & Matsui, 2007). Such features could have significant implications for the delivery of NPs, as accessing hypovascular tumors would be challenging and the differences in vascularization will likely influence NP tumor accumulation.

## 5 ENDOSOMAL ESCAPE

Following tumor extravasation, either drug released from NPs passively diffuse, or NPs themselves (presumed to be larger NPs such as nucleic acids and peptides, see the review Yameen et al., 2014) undergo cellular internalization to exert their therapeutic effect on nuclear targets. Active uptake mechanisms govern the delivery of NPs into tumor cells and these include endocytic pathways such as clathrin‐dependent, caveolin‐dependent, macro‐pinocytosis and phagocytosis. Other endocytosis routes which have been reported entail electroporation, cytoplasmic microinjection and transmembrane penetration (H. Z. Zhang et al., 2015). Endocytosis of NPs involves engulfment in membrane invaginations and formation of intracellular vesicles (endosomes). NPs are then transported in vesicles from early endosomes (pH 7.4) to late endosomes and eventually to lysosomes (pH 5).

The surface charge, size and shape of NPs have proven to be a major determinant of cellular internalization. Several groups have reported heightened internalization of positively charged NPs compared with their negatively charged counterparts in different tumor cell types (Harush‐Frenkel, Rozentur, Benita, & Altschuler, 2008; Kou, Sun, Zhai, & He, 2013). This has been exploited through the fabrication of charge‐conversion NPs which aim to facilitate improved tumor cell entry. The underlying mechanism is to switch the charge of the NP in response to the microenvironment it is exposed to (Cai & Mao, 2016; M. Li et al., 2018). Additionally, NPs smaller than 200 nm were preferentially internalized by clathrin‐dependent endocytosis, while with increasing sizes beyond 200 nm, a shift towards caveolin‐dependent endocytosis was observed (Oh & Park, 2014; Rejman, Oberle, Zuhorn, & Hoekstra, 2004). It should be noted that studies assessing size‐ or charge‐dependent effects often utilize a limited set of materials that differ in ways other than size and charge. Therefore, more data are needed in order to confirm the wider applicability of such physicochemical associations.

As mentioned earlier, active targeting of NPs is also a promising approach to facilitate their internalization. Endocytic pathways may result in trafficking, to either the acidic environment of lysosomes where the NP is subsequently degraded, or a nontarget organelle site. In light of this, recent research has focused on strategies to promote endosomal escape or avoid lysosomes. The incorporation of cationic polymers, such as polyethylenimine and poly‐l‐lysine, in NP design represent a viable strategy for inducing release from endosomal compartments. The cationic charge of the NP interacts with the outer negatively charged surface of the endosomal membrane, leading to membrane flipping and subsequent destabilization, also known as the flip‐flop mechanism (Varkouhi, Scholte, Storm, & Haisma, 2011). Furthermore, pH‐sensitive material can induce endosomal escape via the proton sponge effect. Buffering polymers absorb protons thereby preventing acidification of endosomal vesicles. This ultimately leads to osmotic swelling inside the endosome, membrane rupture and eventual leakage of NPs into the cytosol. Some drugs may take effect instantly, while those that act upon DNA must cross the nuclear membrane in order to exert their therapeutic effect.

## 6 IMPROVING TRANSLATION

The application of nanotechnology in drug delivery is increasing rapidly. However, despite significant advances in materials understanding, clinical translation is still limited. Research is now shifting away from traditional methods that prioritize the drug delivery system, to disease‐based design approaches; a concept where the underlying biology and the material's physicochemical properties are given precedence (Hare et al., 2017). As in the case of CRC liver metastases, a better understanding of the disease pathophysiology and how this can influence accumulation, distribution, retention and efficacy requires further consideration. However, these tumors can be highly heterogeneous and throughout the progression of the disease, intra‐ and inter‐patient variability is apparent (Moro et al., 2018; Sveen et al., 2016; Vermaat et al., 2012). Consequently, certain tumors may be more amenable to NP drug delivery than others, as fewer barriers may impede the disease‐specific localization of NPs. In view of this, the band of fibrotic stroma present in desmoplastic liver metastases is enriched with collagen Type IV and has dense lymphocytic infiltration. Hence the porosity and rigidity of the stroma may further complicate the navigation of NPs towards the tumor cells. Pushing liver metastases patterns demonstrate the most angiogenesis and therefore display a significantly larger size of metastases (Oliveira, Alexandrino, Cipriano, & Tralhao, 2019). Correspondingly, the upregulation of vascular factors may present additional opportunities for active targeting (Dome, Hendrix, Paku, Tovari, & Timar, 2007).

A major issue with the clinical translation of NPs is the gap between preclinical and clinical studies. Currently, preclinical models of CRC liver metastases do not completely recapitulate the different aspects of these tumors or the heterogeneity. The majority of animal models are syngeneic or xenografts and use ex vivo manipulated tumors. Therefore, their ability to mimic CRC liver metastases is limited. Differences in features such as the size/pattern of the tumor, vascularity, levels of IFP and presence of hypoxia highlight some of the challenges of using such models for estimating clinical NP performance. To offset this, various sophisticated in vitro platforms that act as predictive human tissue models are being developed to profile NPs. Examples include three‐dimensional tumor models (spheroids and organoids) consisting of several cell types as well as components of the ECM, which display improved predictive power of in vivo pharmacological efficacy as compared to traditional monolayer cell culture (Durymanov et al., 2019; Fong, Toh, Yu, & Chow, 2017; Langhans, 2018). More recently it was demonstrated that organoids derived from human CRC liver metastases were able to recapitulate additional aspects of the disease (Buzzelli, Ouaret, Brown, Allen, & Muschel, 2018). When applied in vivo, mCRC organoids were competent and colonized within the liver, displaying their potential for translation from in vitro to in vivo preclinical models (Cristobal et al., 2017; Fujii et al., 2016). As CRC metastasis is a multifaceted and heterogeneous disease, merging different organoid models offers the opportunity to study the effect of intratumoral heterogeneity on CRC phenotypes. Furthermore, patient‐derived xenograft animal models which display metastasis are the “go to” models being used to characterize in vivo end points. They are able to mimic tumor cell interactions and generate a relevant tumor microenvironment (T. Jiang, Jin, Liu, & Pang, 2017). A shortcoming of patient‐derived xenograft models is that the immune system is impaired, which impacts cancer progression (Jung et al., 2017). An alternative “go to” approach for developing liver metastasis is the use of genetically engineered mouse models, which have intact immune systems. This method poses challenges in evaluating therapeutic responses, as the metastases have long latency (Young, Hong, Lee, & Cho, 2017). At present there is no ideal animal model for entirely recapitulating the mechanisms and processes found in human CRC liver metastasis. Consequently, multiple models are frequently needed to address specific clinically relevant experimental questions.

More advanced models termed “organ‐on‐a‐chip” systems may be key to accurately assessing the toxicity, pharmacokinetics, and pharmacodynamics of NPs. These microfluidic devices are able to integrate vascular networks, reproduce the tumor microenvironment and have the potential for personalization (Millard et al., 2017). However, the use of these devices for evaluating NPs has not been extensively implemented, meaning that there is not yet enough data to show their predictive power clinically. Nevertheless, the application of more relevant preclinical models in NP testing will likely translate to clinically beneficial outcomes in the medium to long term.

## 7 CONCLUSIONS AND PERSPECTIVES

Current therapeutic options to target CRC liver metastases are limited and the clinical need to deliver tolerable and effective therapeutics to such regions is clear. It is hoped that designing agents to efficiently access the liver and eradicate metastases, will provide a larger survival benefit in comparison to current chemotherapy, which is only of modest efficacy. In addition to CRC, the liver is a common site of metastasis for various types of solid tumors including sarcoma, breast, kidney, pancreatic, ovarian, prostate, and lung cancers (Brodt, 2011; Fan & Gao, 2017; Hess et al., 2006). As a metastatic site, the liver poses significant challenges, which impairs drug distribution and subsequent efficacy. An antimetastatic nanotechnology‐based approach may therefore present a promising route for tackling such issues.

Evidently, successful drug delivery is hampered by the presence of a number of biological and physicochemical barriers, which in turn impairs the ability to effectively treat cancers. Rather than designing nanomedicines which take a holistic approach, that is, addressing each and every stumbling block, the focus should remain on the development of a safe and efficacious drug delivery system. In relation to CRC liver metastases this involves selectively targeting the tumor cells, while simultaneously minimizing nonspecific interactions, be it binding with or uptake into other cell types located within the hepatic sinusoids (B cells, hepatocytes, KCs and LSECs). This may be central to additionally targeting micrometastases which often go undetected and are associated with poor prognosis. Efforts also need to be made to improve extravasation and intratumoral accumulation, both of which may benefit the efficacy of the treatment. Coincidentally, ensuring that the drug is either released so that it diffuses throughout the tumor region, or that the drug is delivered intact to its site of action, are critical factors in assuring efficacy. Although the physicochemical properties of nanomedicines may not be directly involved in the above processes, collectively with the tumor biology they play a key role in determining the interactions and pharmacokinetics of nanomedicines. In view of this, the physicochemical properties of the NP need to be optimized (size, charge, shape, and surface chemistry), making sure to integrate these biological factors into the design of nanomedicines so that they do not impede efficacy.

Other factors such as tumor heterogeneity with respect to the EPR effect and enhanced circulation times are important features to consider, but they do not guarantee that nanomedicines will reach or access the tumor site. Simply relying on the EPR effect is not sufficient for delivery, especially into poorly perfused tumors, for example, replacement liver metastases. Added to this, some studies have indicated that extending circulation time beyond a certain point does not lead to improved efficacy (Nichols & Bae, 2013). Moreover, overexpression and specificity should not be conflated when considering targeting strategies. In particular, precautions should be taken for strategies that aim to reshape the tumor microenvironment. Such methods may promote tumor cell migration and increase drug resistance. For many a target, the intratumoral expression varies both spatially and temporally according to the microenvironmental conditions (Bae & Park, 2011). Hence, better characterization of molecular targets that are specific to CRC liver metastases is essential, with serious consideration being given to whether a targeting strategy will convey actual benefit.

During preclinical development it is vital to understand the well‐described interactions with components of the immunological and hematological systems (Halamoda‐Kenzaoui et al., 2019; Rosslein et al., 2017; Urban, Liptrott, & Bremer, 2019) to de‐risk translation (Halamoda‐Kenzaoui et al., 2019). NPs carrying a cationic charge interact with biological membranes electrostatically, and trigger hemolysis, platelet activation and induction of leukocyte procoagulant activity (Dobrovolskaia, Shurin, & Shvedova, 2016). As well as surface charge, NPs with high aspect ratios have been shown to activate intracellular sensors such as the NLRP3 inflammasome (Baron et al., 2015). Use of surface coatings such as PEG may reduce interactions with immunological systems by preventing opsonization by proteins such as immunoglobulins and components of the complement system. However, it has also been demonstrated that many people produce anti‐PEG antibodies as a consequence of pre‐exposure to PEG‐containing products (B. M. Chen et al., 2016; Richter & Akerblom, 1984). The presence of anti‐PEG antibodies has been correlated with immediate‐type hypersensitivity reactions to a PEGylated aptamer (Pegnivacogin) in a clinical trial (Povsic et al., 2016). Infusion reactions are immune‐mediated toxicities that occur within the first minutes to hours of the systemic administration of various drug products, at their relevant therapeutic doses. Activation of complement and the resultant complement activation‐related pseudoallergy is one mechanism underlying such infusion reactions to NPs (Neun, Barenholz, Szebeni, & Dobrovolskaia, 2018). A thorough review of the safety considerations for NP development is beyond the scope of this review. However, a robust understanding of interactions with immunological and hematological systems is critically important to the translation of NPs for CRC.

Even though there has been a myriad of advances in nanomedicine and drug delivery, there are various concepts and mechanisms that are still not fully understood due to a lack of experimental data. Some questions that still need to be answered include the dynamics underlying the EPR effect clinically within the different liver metastases growth patterns, the importance of biodegradability and clearance of the nanocarrier within the liver metastases from a toxicity perspective, the consequences of long‐term exposure to the NP, and the significance of modulating the different immune cells located in the sinusoids. It may be that patient‐ or cancer‐specific stratification is required to ultimately lead to an enhanced understanding and subsequent improved clinical success. It is apparent that greater insight into the opportunities and challenges presented by cancer nanomedicines is required. To support the successful development and clinical translation of nanomedicines, converging ideas from both nanotechnology and tumor biology is critical. If these challenges can be met, nanotechnology holds great promise for improving patient survival through transforming the paradigm of cancer treatment.

## CONFLICT OF INTEREST

A.O. and S.P.R. are co‐inventors of patents relating to drug delivery and are directors for Tandem Nano Ltd. The authors have declared no other conflicts of interest for this article.

## AUTHOR CONTRIBUTIONS

Usman Arshad: Writing original draft, lead; writing review and editing, lead. Paul A. Sutton: Writing review and editing, supporting. Marianne B. Ashford: Supervision, supporting; writing review and editing, supporting. Kevin E. Treacher: Supervision, supporting. Neill J. Liptrott: Writing review and editing, supporting. Steve P. Rannard: Supervision, supporting. Chris E. Goldring: Supervision, lead; writing review and editing, supporting. Andrew Owen: Supervision, lead; writing review and editing, supporting.

## RELATED WIREs ARTICLE

https://doi.org/10.1002/wnan.1353
